# Characterization of cardiac fibroblast-extracellular matrix crosstalk across developmental ages provides insight into age-related changes in cardiac repair

**DOI:** 10.3389/fcell.2024.1279932

**Published:** 2024-02-16

**Authors:** Luke R. Perreault, Mark C. Daley, Matthew C. Watson, Sagar Rastogi, Ajith Jaiganesh, Elizabeth C. Porter, Breanna M. Duffy, Lauren D. Black

**Affiliations:** ^1^ Department of Biomedical Engineering, Tufts University, Medford, MA, United States; ^2^ Cellular, Molecular and Developmental Biology Program, Graduate School for Biomedical Sciences, Tufts University School of Medicine, Boston, MA, United States

**Keywords:** cardiac fibroblast, extracellular matrix, fibrosis, cardiac development, wound healing

## Abstract

Heart failure afflicts an estimated 6.5 million people in the United States, driven largely by incidents of coronary heart disease (CHD). CHD leads to heart failure due to the inability of adult myocardial tissue to regenerate after myocardial infarction (MI). Instead, immune cells and resident cardiac fibroblasts (CFs), the cells responsible for the maintenance of the cardiac extracellular matrix (cECM), drive an inflammatory wound healing response, which leads to fibrotic scar tissue. However, fibrosis is reduced in fetal and early (<1-week-old) neonatal mammals, which exhibit a transient capability for regenerative tissue remodeling. Recent work by our laboratory and others suggests this is in part due to compositional differences in the cECM and functional differences in CFs with respect to developmental age. Specifically, fetal cECM and CFs appear to mitigate functional loss in MI models and engineered cardiac tissues, compared to adult CFs and cECM. We conducted 2D studies of CFs on solubilized fetal and adult cECM to investigate whether these age-specific functional differences are synergistic with respect to their impact on CF phenotype and, therefore, cardiac wound healing. We found that the CF migration rate and stiffness vary with respect to cell and cECM developmental age and that CF transition to a fibrotic phenotype can be partially attenuated in the fetal cECM. However, this effect was not observed when cells were treated with cytokine TGF-β1, suggesting that inflammatory signaling factors are the dominant driver of the fibroblast phenotype. This information may be valuable for targeted therapies aimed at modifying the CF wound healing response and is broadly applicable to age-related studies of cardiac remodeling.

## Introduction

Although cardiomyocytes (CMs) function as the electrical and contractile units of the myocardium, the maintenance and turnover of the cardiac extracellular matrix (cECM) is primarily the responsibility of cardiac fibroblasts (CFs). As primary mediators of the cECM, a crosstalk between CFs and cECM plays a critical role in their mutual functions. The extracellular matrix provides structural support and mechanical cues to resident cells, in addition to storing bioactive signaling molecules. Fibroblasts remodel the cECM via the deposition of matrix proteins and the release of matrix metalloproteinases (MMPs), proteases that break down cECM components, subsequently liberating cytokines, growth factors, and bioactive cECM fragments (e.g., matrikines and matricryptins) from the structure of the cECM (House et al., 2003; Souders et al., 2009; Shinde and Frangogiannis, 2014; Sadri et al., 2022; Reese-Petersen et al., 2023). Importantly, this dynamic relationship undergoes significant alterations with developmental age as cECM composition and fibroblast behavior gradually change. The synthesis and expression of critical cECM components like collagen and fibronectin change with development (Mays et al., 1991; Terracio et al., 1991; Farhadian et al., 1995). Concomitantly, CF behavior changes over time, with proliferative capability reducing with age, in addition to alterations in integrin expression and the expression and activity of MMPs and tissue inhibitors of MMPs (TIMPs) (Lindsey et al., 2005; Perreault et al., 2021; Magaud et al., 2023).

Age-related changes in cardiac tissue maintenance contribute to major alterations in wound healing and tissue repair. Fibrosis, the excessive deposition of noncompliant, collagenous tissue by CFs to rapidly stabilize necrotic tissue, is observed in adult cardiac tissues after damage but reduced in fetal and neonatal mammals. This may be due partly to the transient ability of young (e.g., fetal and neonatal) developmental-age CMs to proliferate up to a week post-birth, facilitating the regenerative healing of the myocardium (Porrello et al., 2011; Haubner et al., 2012; Williams et al., 2014; Haubner et al., 2016; Sun et al., 2023). Indeed, some studies have indicated that this juvenile repair capability can produce near-complete functional cardiac restoration after a myocardial infarction (MI) (Haubner et al., 2012; Agrawal, 2022). This is in sharp contrast to what is seen in adult cardiac tissue: when destabilized by injury, environmental stimuli (e.g., cytokine transforming growth factor β1 or TGF-β1 (Brown et al., 2005; Aránguiz-Urroz et al., 2011; Biernacka et al., 2011; Kawaguchi et al., 2011; Gao et al., 2016; Cho et al., 2018)) induce CF transition to a myofibroblast phenotype (Cho et al., 2018).

Myofibroblasts are characterized by increased proliferation, mobility, and contractility due, in part, to their upregulation of alpha-smooth muscle actin (α-SMA). This transition is mediated via TGF- *ß* receptors on the CF cell surface: TGF-β1 binds to these receptors, triggering intracellular mediators of TGF-β1 signaling, SMAD2 and SMAD3 (Moustakas, 2002). SMADs 2/3 are phosphorylated and generate a complex with SMAD4, entering the nucleus and altering gene expression, facilitating the CF phenotypic transition (Moustakas, 2002; Ma et al., 2012; Wu et al., 2018). Myofibroblasts drastically upregulate cECM protein production during the repair of damaged tissue, particularly collagen I. Although this cECM production is initially necessary to stabilize the necrotic heart wall, persistent deposition in adult tissue leads to fibrosis, impeding heart function due to tissue stiffening and reduced electrical conduction (Svystonyuk et al., 2015; Cho et al., 2018; Pakshir and Hinz, 2018; Brassington et al., 2022). CFs are highly sensitive to alterations in tissue mechanics, and increased stiffness has been shown to drive CF activation, inducing a feedback loop in which a persistent myofibroblast phenotype is generated (Hinz, 2009; Herum et al., 2017). This problem translates to challenges in culturing these cells *in vitro*, wherein stiff culture plastic can similarly induce CF activation. Conversely, soft culture substrates and hydrogels appear to attenuate mechanically induced CF activation in culture (Walker et al., 2021; Ebrahimighaei et al., 2022).

There is mounting evidence that age-dependent changes in tissue maintenance are tied to concurrent changes in ECM composition and CF behavior. *In vitro* studies have shown that juvenile cECM can induce increased cell expansion compared to adult-sourced cECM (Williams et al., 2014; Wang et al., 2019), and the work by Li et al. indicated that engineered cardiac tissues have greater electrical and mechanical function when seeded with fetal CFs compared to adult CFs (Li et al., 2017). Furthermore, RNA sequencing of cardiac fibroblasts at fetal, neonatal, and adult developmental ages performed in our laboratory has suggested the CF inflammatory response is strongly age-dependent, with immune/inflammatory genes being upregulated in postnatal day 1 CFs compared to fetal CFs (Perreault et al., 2021). Taken together, these studies reinforce the notion that developmental age can drive changes in cell behavior and cardiac function. However, none of these studies explored whether age-dependent changes in ECM and CFs may act synergistically.

In this study, we investigated the relationship between CFs and cardiac ECM at fetal, neonatal, and adult developmental ages, studying whether age-dependent CF–ECM interactions may attenuate the CF transition toward activation when stimulated with TGF-β1. We evaluated the CF migration rate, stiffness (which is positively correlated with CF activation (Brown et al., 2007; Mitchell et al., 2007; Justus et al., 2014)), cell morphology, and cell expression of α-SMA using imaging and examined protein expression of α-SMA, SMAD2, and SMAD3 using Western blot. We observed that cECM age does contribute in part to activation but is highly variable with respect to CF developmental age: both mechanical and biochemical CF responses to cECM exhibited significant differences across developmental ages, but upon application of TGF-β1, age-dependent variance is diminished. This suggests that TGF-β1 may dominate CF activation over the developmental state of cECM, regardless of treatment.

## Methods

### Heart harvest from fetal, neonatal, and adult rats

All animal procedures were performed in accordance with the Institutional Animal Care and Use Committee at Tufts University and NIH Guide for the Care and Use of Laboratory Animals. Pregnant Sprague–Dawley rats (∼3 months old) were deeply anesthetized with 3%–5% isoflurane and euthanized via heart removal. Fetal pups (embryonic day 18) were isolated from the uterus, euthanized by conscious decapitation, and hearts were isolated. Adult Sprague–Dawley male rats (∼7 weeks old) were euthanized for heart harvest using the same method as pregnant dams. Neonatal pups (postnatal day 1) were euthanized by conscious decapitation prior to heart harvest. Freshly isolated hearts were stored in an ice-cold solution of sterile PBS with 20 mM glucose and immediately used for cell isolation, as described below.

### Primary cardiac fibroblast isolation and culture

Cardiac fibroblasts were isolated from fetal, neonatal, and adult rat hearts, following previously described methods (Au - Ye et al., 2011; Williams et al., 2014; Gao et al., 2016). After euthanasia and heart harvest, left ventricles from adult rats were separated from the heart and minced to pieces less than 1 mm^3^ in size. Fetal and neonatal hearts were minced whole. Minced tissue underwent 3–5 serial digestions of collagenase type 2 (Worthington Biochemical Corporation, Lakewood, NJ) in sterile PBS with 20 mM glucose, passed through a 40-μm cell strainer, and centrifuged. Cells were counted using a hemocytometer and either plated immediately for experiments with P0 cells (described below) or plated for expansion. In both cases, cells were pre-plated for 1 h in DMEM (Gibco) with 10% fetal bovine serum (Gibco), 1% penicillin–streptomycin (Invitrogen), and 0.1 mM ascorbic acid (Sigma-Aldrich), followed by the removal of nonadherent cells and the reapplication of media (Gharaibeh et al., 2008; Gao et al., 2016).

### Decellularization and solubilization of cardiac ECM

Both fetal and adult pig hearts were used for decellularization and cECM isolation. Pig cECM was selected due to its ability to isolate fetal cECM in large quantities (versus fetal rats) and the overall conservation of cardiac matrix proteins across mammalian species (Badylak, 2004). Ventricles from fetal pig hearts (7–9 inches in full length) (Nebraska Scientific) were cut into ∼1-cm^3^ pieces and decellularized with 0.5% SDS (Thermo Fisher Scientific) in DI water for 1–2 days, until samples appeared white. Samples were further processed with Triton X-100 (Sigma-Aldrich) at 0.5% in DI for 1 h, followed by rinsing in multiple changes of DI water for 48 h to remove the remaining detergent. Adult pig left ventricle tissue was obtained from the local abattoir and decellularized using the same protocol but with 1% SDS and Triton X-100 instead of 0.5%. ECM coatings for cell culture studies were prepared as previously described (Williams et al., 2014). In brief, decellularized samples were frozen at −20°C and lyophilized before being milled into a fine powder. This ECM powder was solubilized via pepsin digestion in 0.1 M HCl for 12 h (6 h for fetal ECM), adjusted to pH 7.4, and lyophilized a second time. Digested ECM was reconstituted to 20 mg/mL in DI water immediately prior to use.

### Measurement of cardiac fibroblast stiffness via atomic force microscopy

Fetal, neonatal, and adult Sprague–Dawley rat CF cell stiffness was measured by atomic force microscopy (AFM), using a Veeco Dimension 3100 AFM instrument. Cells used were between passages P1 and P3 and were seeded onto 2-cm^2^ glass coverslips (coated with 50 ug/cm^2^ adult porcine cECM, 50 ug/cm^2^ fetal porcine cECM, or a combination of 0.02% (wt/vol) gelatin and 0.5% (wt/vol) fibronectin, for a total of three measured cells per group). Stiffnesses for 3 cells per experimental group were measured using Novascan borosilicate glass particle cantilevers with a 5-µm-diameter bead and a rated spring constant value of 0.06 N/m. For each cell, a 5-μm^2^ region was analyzed using the contact mode on the AFM, and a minimum of 32 discrete measured points were acquired for each region (out of a maximum of 265). Hertzian theory (Fischer-Cripps, 1999) was used to calculate Young’s modulus for each indent curve over the entire measured 2D force volume, using a custom MATLAB code to average measured values for each sample. With these data, we were able to determine differences in cell stiffness with respect to cECM coating developmental age and cell developmental age.

### Scratch assay analysis of the cardiac fibroblast migration rate

Fetal, neonatal, and adult Sprague–Dawley rat CFs between passages P1 and P3 were plated in a 24-well plate coated with 50 ug/cm^2^ of adult porcine cECM, 50 ug/cm^2^ fetal porcine cECM, or a combination of 0.02% gelatin and 0.5% fibronectin at a density of 50,000 cells/cm^2^ in serum-containing media for 24 h (N = 6 wells per group). Following seeding, cells underwent serum starvation overnight in 50:50 DMEM:Ham’s F12 Nutrient Mix (Gibco), supplemented with 0.5% insulin transferrin selenium (Corning), 0.2% BSA (Thermo Fisher Scientific), 0.1 mM ascorbic acid, and 1% penicillin/streptomycin, hereafter referred to as serum-free media.

After starvation, the serum-free media was aspirated, and a P200 pipette tip was used to generate a scratch across the diameter of each well, yielding an empty ∼1-mm-thick region in the center of each well. Cells were rinsed in PBS, and serum-free media was reapplied. Plates were then transferred to a live-cell stage on a Keyence BZ-X8700 series microscope. Three regions per well were evaluated and imaged sequentially using a z-stack. All wells were sequentially imaged for at least 12 h with a maximum interval of 30 min (timing varied slightly due to stage auto-focus, tracking, and a set time to allow media fluid motion to settle).

Images were loaded into Keyence Image Analysis software to generate a time-lapse video. Keyence Image Analysis software was used to automatically identify the best time lapse taken from each z-stack (i.e., most free from image artifacts or cell debris), which was then used to analyze cell migration for that sample. Time-lapse images were imported into ImageJ to determine the total area taken up by cells in successive images. These data were then evaluated in MATLAB using area data calculated in ImageJ to determine the rate of closure, which is considered here to be consistent with the migration rate, measured as the percentage of closure per hour. The average rate of closure for each group (assuming a linear fit) was then determined.

### Fibroblast imaging, proliferation, and activation measurements

After isolation and pre-plating, P0 CFs were seeded in a 24-well plate at a density of 50,000 cells/cm^2^. The cells were cultured with 50 ug/cm^2^ adult porcine cECM, 50 ug/cm^2^ fetal porcine cECM, or a combination of 0.02% gelatin and 0.5% fibronectin in serum-containing media. After 24 h, serum starvation was performed overnight in serum-free media (described above).

After 24 h of serum starvation, CFs were either activated to a myofibroblast phenotype with 2.5 ng/mL transforming growth factor β1 (TGF-β1, PeproTech) or left untreated. The application of TGF-β1 was considered Day 0 for culture experiments. On days 0 and 3, samples were fixed in ice-cold 4% paraformaldehyde for 10 min at room temperature. Cells were permeabilized in 0.05% Triton X-100 in PBS for 10 min and blocked with 5% donkey serum and 1% bovine serum albumin (BSA) in PBS overnight at 4°C. Cells were stained overnight with 0.5 μg/mL of the rabbit anti-Ki67 antibody (Abcam, ab15580) and mouse anti-α-SMA antibody (Abcam; ab7817 and ab119952, respectively) at 4°C. Next, donkey anti-mouse 568 (Invitrogen) and donkey anti-rabbit 488 were applied at a 1:400 dilution in BSA for 2 h. The nuclei were stained with DAPI during the last 30 min. Samples were then washed three times with PBS containing Tween (PBST) and imaged using a Keyence BZ-X8700 series microscope. Images were analyzed using a custom CellProfiler pipeline (Broad Institute) that counted the total number of cells per sample and the number of Ki67^+^ (proliferative) cells and determined the percentage of myofibroblasts to fibroblasts based on the fluorescence intensity of α-SMA (cells with fluorescence intensity above an arbitrary threshold, based on Day 0 α-SMA fluorescence intensity, were recorded as myofibroblasts in the CellProfiler pipeline).

### Western blot analysis of cardiac fibroblasts on Day 3 of cell culture

Fetal, neonatal, and adult rat CFs (between P2 and P4) were cultured on fetal cECM, adult cECM, or gelatin-fibronectin in T25 flasks (N = 4 flasks per group) in serum-free media. After 3 days in serum-free media, cells were rinsed in cold PBS before application of ice-cold lysis buffer containing 1x NP40 (Alfa Aesar), 0.025% (w/v) sodium deoxycholate (Sigma-Aldrich), 200 µM sodium orthovanadate (Sigma-Aldrich), and 1x protease inhibitor cocktail (Sigma-Aldrich) for 5 min at 4°C. Lysates were sonicated for 15 s on ice and kept on ice for an additional 30 min with frequent agitation. Samples were then sonicated a second time for an additional 30 s before centrifugation at 13,000 rpm at 4°C for 15 min. Insoluble protein pellets were removed, and the supernatant was retained. Protein concentration was then measured using a Pierce BCA Protein Assay Kit, following the manufacturer’s protocol (Thermo Fisher Scientific).

Equal amounts of protein (15 µg) were run on 4%–12% pre-cast TruPAGE (Sigma-Aldrich) gels and transferred to a nitrocellulose membrane using manufacturer protocols. Membranes were blocked for 3 hours at room temperature in a 5% (w/v) powdered milk solution in Tris-buffered saline with 0.1% Tween-20 (TBST). After three rinses in TBST, primary antibodies were diluted 1:1000 in 5% bovine serum albumin in TBST and applied overnight at 4°C. Assayed proteins included α-SMA (anti-mouse, Abcam, ab7817), SMAD2/3 (anti-rabbit, Cell Signaling Technology, #8685), and GAPDH (anti-rabbit, Cell Signaling Technology, #2118). Blots were rinsed in TBST three times, followed by incubation with an HRP-conjugated secondary antibody (Thermo Fisher SA1-200) diluted 1:1000 in 5% milk in TBST for 2 h at room temperature. Finally, an additional set of three TBST rinses was performed prior to imaging. Images were developed on a G: BOX Chemi XR5 system (Syngene) using the Amersham ECL Prime Western Blotting Detection Reagent (GE Healthcare). Stripping was performed no more than twice on each blot using Restore Western Blot Stripping Buffer (Thermo Scientific). Densitometry was performed in ImageJ. Protein bands were row-normalized, and proteins of interest were normalized to GAPDH.

### Statistical analysis

Statistical significance was determined using dimensionally appropriate analysis of variance (ANOVA) tests, followed by Tukey’s multiple comparisons test in GraphPad Prism. Results were considered statistically significant at *p* < 0.05. All statistics are reported as the average ± standard deviation, unless otherwise noted.

## Results

### Cardiac fibroblast cell stiffness is sensitive to the developmental age of cECM coating

**FIGURE 1:**
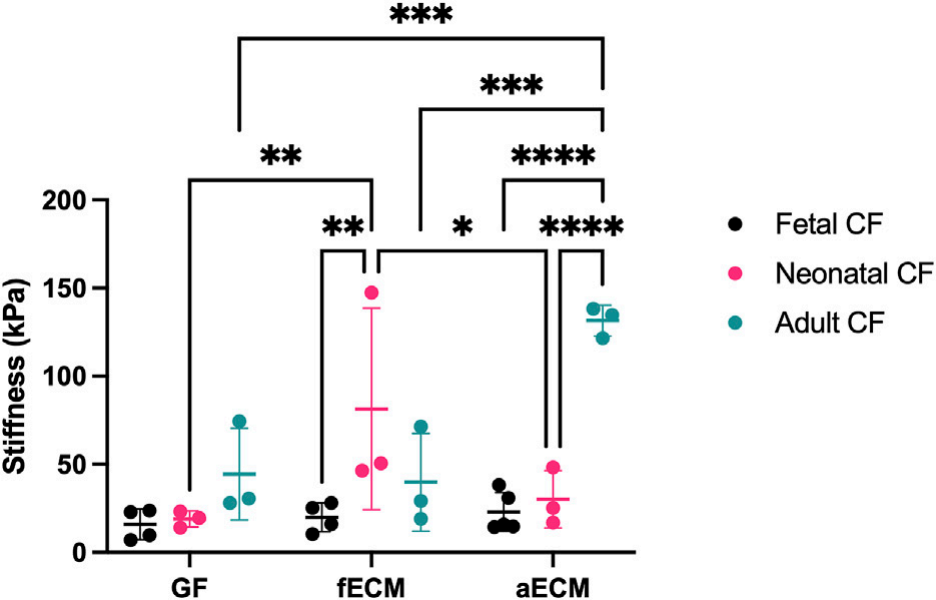
AFM data of fetal, neonatal, and adult cardiac fibroblasts (N ≥ 3 for all groups). GF, gelatin–fibronectin; fECM, fetal cardiac ECM; aECM, adult cardiac ECM. *, *p* < 0.05; ** *p* < 0.01; ***, *p* < 0.001; ****, *p* < 0.0001.

To evaluate whether the CF developmental age and the developmental age of cECM impact CF mechanics, we performed AFM to directly evaluate cell stiffness (Figure 1). AFM measurements were conducted assuming an increase in stiffness correlated with an increase in cytoskeletal protein formation and structure, particularly with respect to α-SMA, which is upregulated when CFs undergo transition to a myofibroblast phenotype. We hypothesized that solubilized fetal cECM might attenuate an increase in CF stiffness compared to solubilized adult cECM.

All three CF developmental ages possessed similar stiffnesses on the age-independent gelatin–fibronectin control substrate (fetal CFs: 15.87 ± 8.68 kPa; neonatal CFs: 18.98 ± 4.60 kPa; and adult CFs: 44.36 ± 26.03 kPa). Compared to the gelatin–fibronectin control substrate, the stiffness of fetal CFs was unchanged by either fetal cECM (19.94 ± 8.16 kPa) or adult cECM (22.89 ± 11.03 kPa). Contrastingly, neonatal CFs possessed a significantly higher stiffness on fetal ECM (81.49 ± 57.18 kPa; *p* < 0.05), although this average was skewed by an outlier at 147.47 kPa, and adult CFs possessed the largest stiffness of any group when cultured on adult ECM (131.56 ± 8.76 kPa; *p* < 0.05).

### Scratch assay analysis suggests CF migration rate informed by cECM developmental age

**FIGURE 2:**
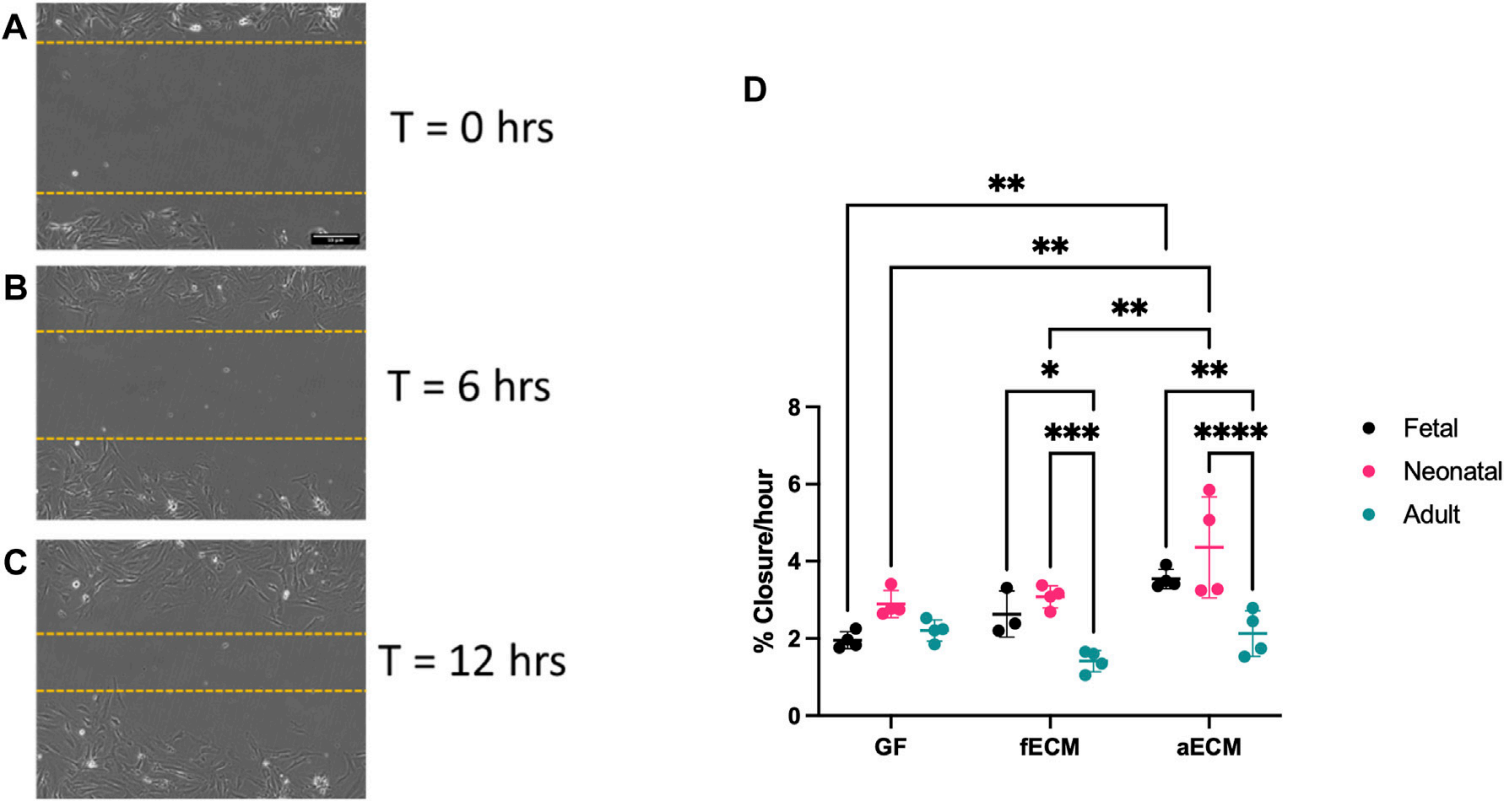
**(A–C)** Representative images of scratch assay used for analysis at 0–12 h. **(D)** Scratch assay data of fetal, neonatal, and adult CFs on gelatin–fibronectin (GF), fetal cardiac ECM (fECM), and adult cardiac ECM (aECM) coatings. N = 3 for all groups. *, *p* < 0.05; **, *p* < 0.01; ***, *p* < 0.001; ****, *p* < 0.0001.

CF migration (Figure 2; Supplementary Figure S1) plays a critical role in the cardiac wound healing response, with increased migration being indicative of CF activation and transition to a myofibroblast phenotype. Consequently, we endeavored to evaluate whether the CF developmental age or soluble cECM developmental age impacted the CF migration rate in a scratch assay wound healing model. We hypothesized that CFs and cECM sourced from fetal cardiac tissue would yield the lowest migration rates due to the reduced fibrosis typically observed in the fetal cardiac wound environment (Porrello et al., 2011; Williams and Black, 2012).

All CF developmental ages possessed similar migration rates on control gelatin–fibronectin substrates (Figure 2D) (fetal CFs: 1.96% ± 0.22%/hr; neonatal CFs: 2.89% ± 0.35%/hr; and adult CFs: 2.21% ± 0.28%/hr). When cultured on adult cECM, both fetal (3.54% ± 0.25%/hr; *p* < 0.01) and neonatal CFs (4.36% ± 1.31%/hr; *p* < 0.01) had significantly higher migration rates compared to their gelatin–fibronectin controls. In contrast, we observed no significant differences in the migration rate of adult CFs with respect to the ECM developmental age (gelatin–fibronectin: 2.21% ± 0.28%, fetal: 1.41% ± 0.28%, and adult: 2.13% ± 0.59%). When examining CF migration rates within cECM types, the migration rate of adult CFs was also significantly slower compared to both neonatal and fetal CFs on fetal and adult ECM (Figure 2D). Taken together, this suggests that adult cECM may induce faster migration in young developmental-age CFs compared to gelatin–fibronectin.

### Image analysis indicates proliferation and activation are variable with respect to the cell and cECM developmental age

**FIGURE 3:**
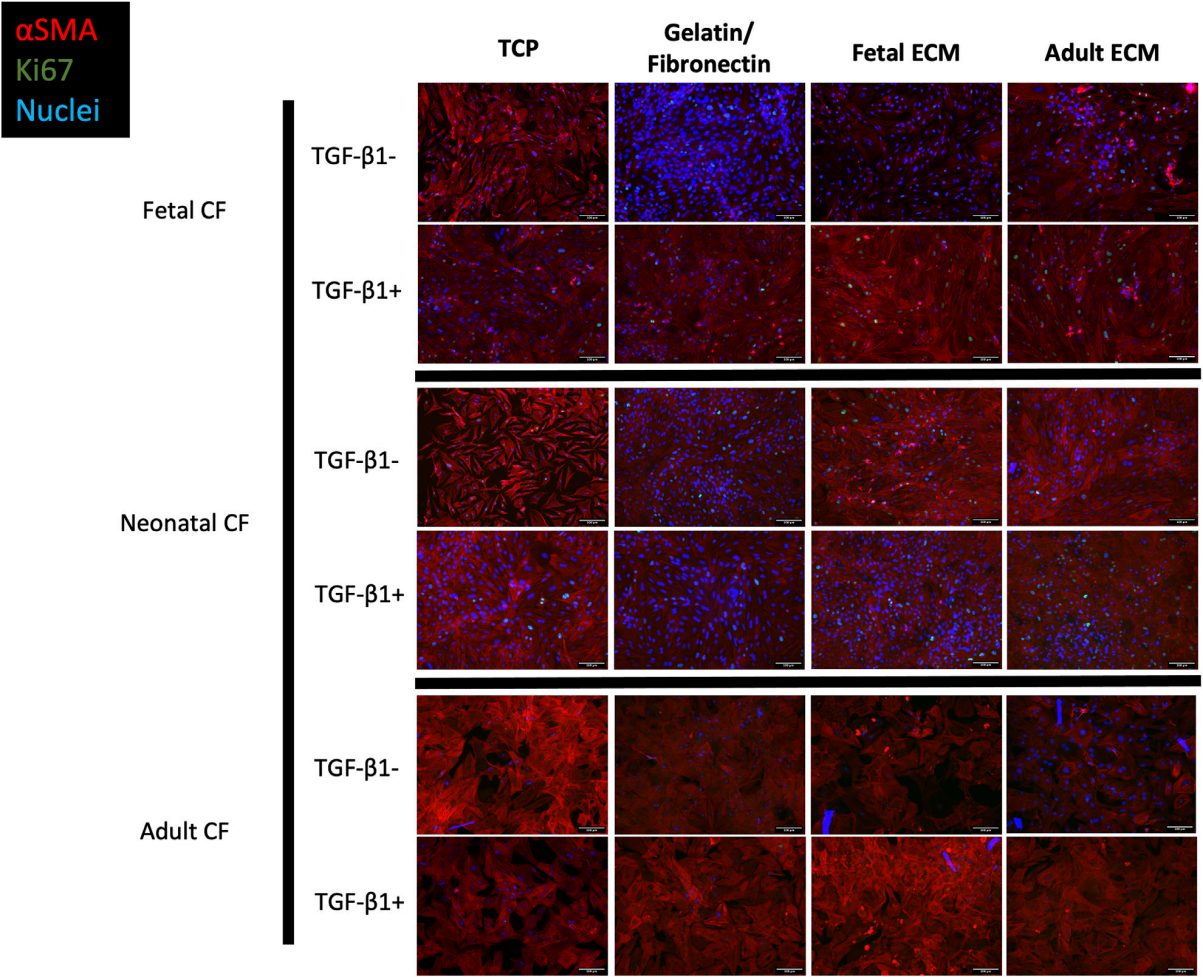
Immunofluorescence imaging of fetal, neonatal, and adult cardiac fibroblasts treated with TGF-β1 and left untreated. Cells were cultured on TCP, gelatin–fibronectin, fetal ECM, or adult ECM. Red, SMA; green, Ki67; blue, DAPI. N > 5 for all groups. Scale bars = 100 um.

To investigate whether TGF-β1-mediated CF activation varied with respect to the CF developmental age or cECM developmental age, we cultured fetal, neonatal, and adult CFs on fetal cECM, adult cECM, gelatin-fibronectin, or uncoated TCP. We then either treated these cells in serum-free media with 2.5 ng/mL of TGF-β1 or left them untreated to determine the impact of cell and cECM developmental age on their activation. We hypothesized that fetal CFs may demonstrate less differentiation to a myofibroblast phenotype and that fetal cECM may attenuate TGF-β1-associated activation and proliferation for all cell developmental ages. We analyzed cultures via immunofluorescence to assess proliferation via Ki67 expression and the myofibroblast phenotype via α-SMA expression, imaging cells on Day 3 of cell culture (Figure 3).

**FIGURE 4:**
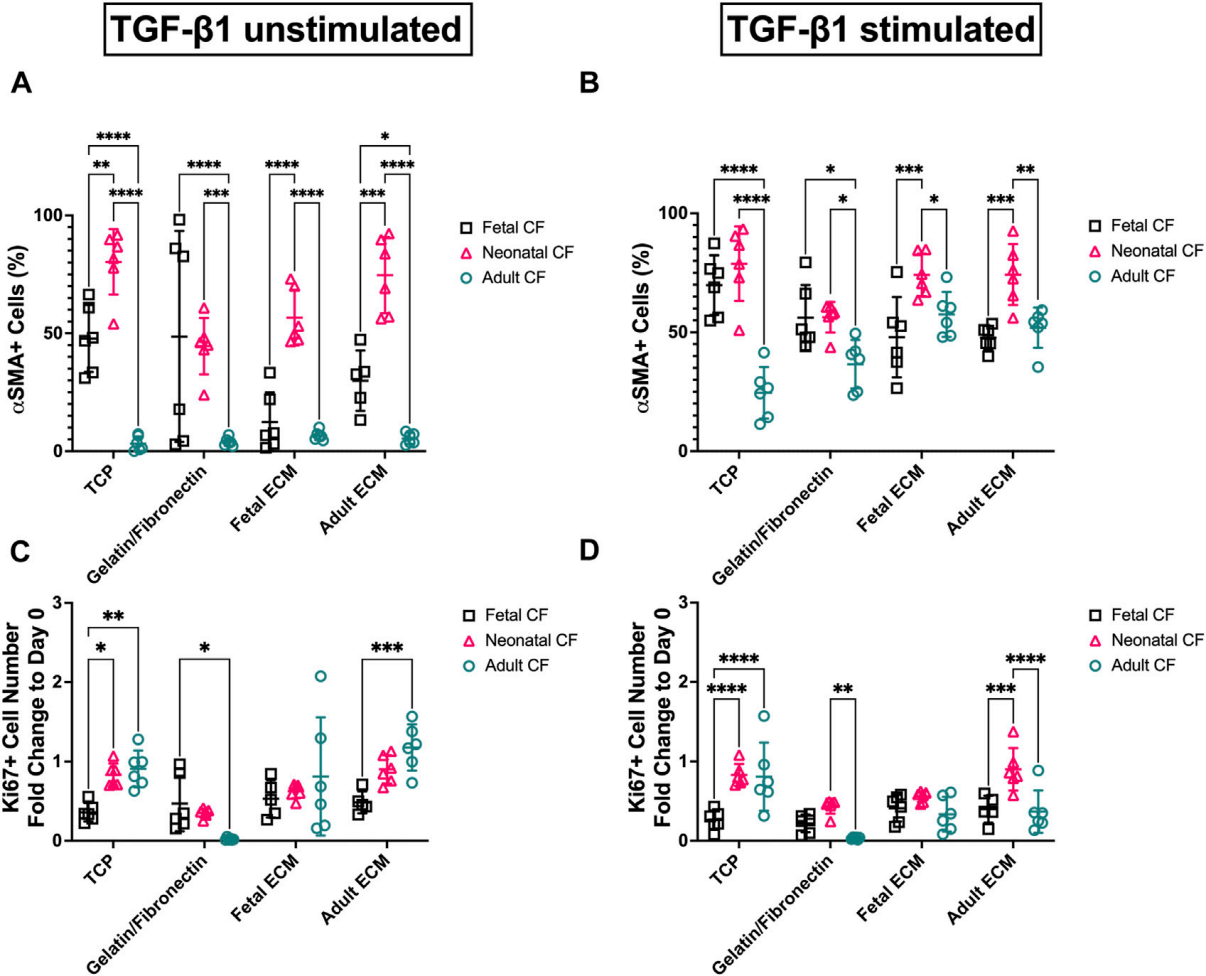
Image quantification of D3 fetal, neonatal, and adult cardiac fibroblasts when cultured on gelatin–fibronectin, fetal ECM, adult ECM, and TCP. **(A)** Fibroblast (untreated) percentage of α-SMA positive cells. **(B)** TGF-β1-treated cardiac fibroblast percentage of α-SMA-positive cells. **(C)** Fold change vs. Day 0 of Ki67 in untreated fibroblasts. **(D)** Fold change vs Day 0 of Ki67 in TGF-β1-treated cardiac fibroblasts. N > 5 for all groups. *, *p* < 0.05; **, *p* < 0.01; ***, *p* < 0.001; ****, *p* < 0.0001. ECM-to-ECM comparisons are also evaluated and reported with significance in Supplementary Table S1.

Morphologically, cells activated with TGF-β1 reflected the conventional phenotype associated with myofibroblasts, including large, elongated α-SMA striations, compared to unactivated cells, which appeared to have lower α-SMA expression coupled with a more rounded morphology. We further analyzed these images using a CellProfiler pipeline to quantify these observed differences in Ki67 and α-SMA expression (Figure 4). For clarity, Figure 4 only presents CF-to-CF age comparisons (i.e., fetal CFs to adult CFs, on fetal ECM). ECM-to-ECM comparisons (i.e., fetal CFs on adult ECM vs fetal CFs on fetal ECM) are also reported here and presented in Supplementary Table S1. For unstimulated CFs, α-SMA expression varied extensively with respect to CF developmental age (Figure 4A; Supplementary Table S1). Adult cardiac fibroblasts presented significantly lower α-SMA expression when cultured on TCP (3.21% ± 3.10%) and gelatin–fibronectin (3.98% ± 1.68% positive α-SMA cells) compared to neonatal (78.85% ± 15.65% on TCP and 56.25% ± 6.42% on gelatin–fibronectin) and fetal CFs (69.84% ± 12.53% on TCP and 56.12% ± 13.76% on gelatin–fibronectin) cultured on the same coatings (*p* < 0.0001). Neonatal CFs presented significantly (*p* < 0.0001) higher α-SMA expression compared to fetal and adult CFs on fetal cECM (56.66% ± 11.83%) and adult cECM (74.69% ± 16.13%, respectively). Neonatal CFs exhibited lower α-SMA fluorescence on gelatin–fibronectin than on TCP (*p* < 0.01). Fetal CFs presented with significantly less α-SMA fluorescence on fetal cECM than on TCP or gelatin fibronectin (*p* < 0.01). Adult CF α-SMA expression did not appear to be affected by coating developmental age.

Activating cells with TGF-β1 appeared to reduce some of these differences in α-SMA expression (Figure 4B; Supplementary Table S1). On TCP, adult CFs exhibited significantly less α-SMA fluorescence (24.51% ± 10.85%) than fetal or neonatal CFs on the same coating (*p* < 0.0001). Adult CF α-SMA expression appeared to be influenced by cECM coating: they exhibited lower expression on TCP compared to adult cECM (51.90% ± 8.47%, *p* < 0.001) and fetal ECM (57.52% ± 9.48%, *p* < 0.0001). Fetal CFs showed significantly lower α-SMA expression than neonatal CFs on fetal cECM (47.91% ± 16.87% vs 74.15% ± 8.90%, respectively, *p* < 0.001) and adult cECM (47.65% ± 5.02% vs 74.24% ± 12.78%, respectively, *p* < 0.001).

Ki67 expression was used as a metric of proliferation and reported as a fold change versus Day 0 to account for potential variability in cell seeding. Differences in both TGF-β1-treated and untreated groups appeared to be minimal, with inconsistent differences in proliferation observed. This is supported by the data in Supplementary Figure S2, which present changes in overall cell density (in cells/mm^2^) across the time course of the experiment. This figure indicates that the overall cell density had a broad range between ∼2000 cells/mm^2^ and ∼8000 cells/mm^2^ on Day 3, which likely had an effect on the quantification of the proliferative cell number. However, untreated adult CFs showed a significantly (*p* < 0.0001) lower fold change in proliferation on gelatin–fibronectin (0.022 ± 0.015) compared to adult CFs cultured on other coatings (Figure 4C; Supplementary Table S1). Furthermore, on adult cECM, fetal CFs showed a lower fold change in proliferation compared to adult CFs (0.48 ± 0.14 vs 1.18 ± 0.29, *p* < 0.001). Analysis of CFs treated with TGF-β1 was inconsistent, but it appeared that many groups had a measureable decrease in proliferative cells versus Day 0 (fold change <1) (Figure 4D; Supplementary Table S1). On TCP, fetal CFs showed a lower (*p* < 0.0001) fold change in proliferation (0.26 ± 0.12) than neonatal and adult CFs. Conversely, on adult cECM, neonatal CFs showed a significantly (*p* < 0.001) higher fold change in proliferation (0.90 ± 0.27) compared to fetal and adult CFs. Taken together, these data do not appear to represent specific trends in CF activation differences with respect to either cell age or cECM developmental age but suggest that CF response is highly variable with respect to both these metrics. However, variability appeared to reduce when CFs were treated with TGF-β1.

### Application of TGF-β1 appears to drive the protein expression of SMAD2/3 and α-SMA in fetal, neonatal, and adult CFs in comparison to the cECM developmental age

To more directly evaluate whether cECM developmental age and/or CF developmental age had impacts on fibroblast response to TGF-β1, we performed Western blot analysis to evaluate CF protein expression of SMAD2/3 and α-SMA. We hypothesized that if cECM or CF developmental age impacted TGF-β1-associated CF activation, SMAD2/3 and α-SMA would both be reduced, indicating a reduction in CF-to-myofibroblast differentiation (Moustakas, 2002; Cushing et al., 2008).

**FIGURE 5:**
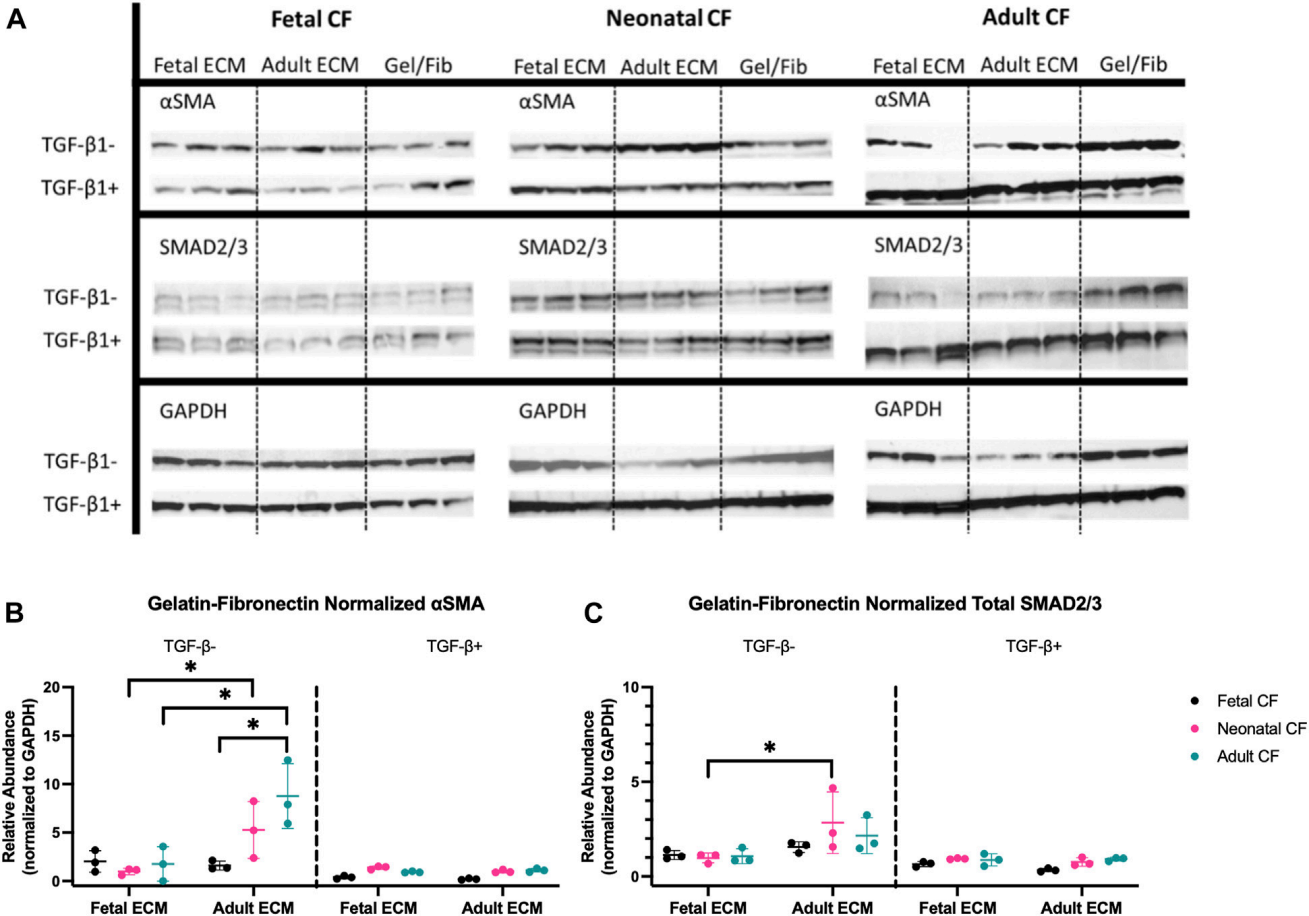
Western blot analysis of fetal, neonatal, and adult cardiac fibroblasts. **(A)** Western blot bands for all tested fibroblast ages cultured on fetal cECM, adult cECM, and gelatin–fibronectin treated with TGF-β1 and those left untreated. **(B)** Relative abundance of α-SMA in both TGF-β1-treated and untreated cardiac fibroblasts, normalized to GAPDH; groups normalized to the gelatin–fibronectin group. **(C)** Relative abundance of total SMAD2/3 in both TGF-β1-treated and untreated cardiac fibroblasts, normalized to GAPDH; groups normalized to the gelatin–fibronectin group. N = 3 for all groups. *, *p* < 0.05, differences in the cECM type are significant for the tested CF age. Bars (*p* < 0.05) indicate significance between cell ages cultured on adult cECM.

Western blotting indicated significant differences in both SMAD2/3 and α-SMA abundance, both with respect to CF developmental age and cECM developmental age (Figure 5A). The data shown are normalized to GAPDH and additionally normalized to gelatin–fibronectin values as a control (Figures 5B, C). Supplementary Figure S3 presents these results without additional normalization to gelatin–fibronectin for comparison. When cells were treated with TGF-β1, neither CF nor cECM developmental age had a statistically significant effect on the abundance of SMAD2/3 or α-SMA with respect to gelatin–fibronectin (Figures 5B, C). This stood in sharp contrast to the results of cells not treated with TGF-β1, which produced significant differences with respect to both the developmental age of cECM and CFs. Both adult and neonatal CFs had significantly higher expression of α-SMA on adult cECM compared to when they were cultured on fetal cECM (*p* < 0.05). Additionally, fetal CFs showed significantly lower α-SMA on adult cECM than adult CFs (*p* < 0.05), suggesting a CF-age-dependent difference in cECM sensing (Figure 5B). SMAD2/3 levels did not appear to be impacted by cECM or CF developmental age, although neonatal CFs expressed higher levels of SMAD2/3 on adult cECM compared to fetal cECM (*p* < 0.05) (Figure 5C). Taken together, the results appear to suggest that fetal cECM might attenuate α-SMA expression in neonatal and adult CFs, and fetal CFs express less α-SMA on adult cECM than adult CFs. These effects are secondary to the impact of TGF-β1 on the cell phenotype and function.

## Discussion

### Cardiac fibroblast mechanical properties show variance with respect to CF and cECM developmental age

Much of the research on evaluating the function of CFs and understanding how their microenvironment contributes to their characteristics and behavior has primarily focused on their mechanical environment. It is well-established that CF culture on stiff substrates can significantly alter the CF phenotype, driving a transition toward activation and differentiation into a myofibroblast (Zhang et al., 2001; Jester et al., 2002; Avery et al., 2018; Cho et al., 2018). This study is novel in contributing a perspective on how soluble cECM fragments may contribute to the cardiac fibroblast phenotype and by further elucidating how CFs differ in the migration rate and stiffness with respect to developmental age.

Contrary to our initial hypothesis, we observed an increased migration rate in fetal CFs compared to adult CFs, particularly on adult cECM (Figure 2). This may be due to differences in the activity levels of the cells in question. However, a study by Chen et al in 1989 evaluated fetal and adult human dermal fibroblasts in culture and tracked their migration rate and metabolic activity. The study concluded that fetal cells exhibited significantly higher migration than their adult counterparts due, in part, to increased metabolic activity and production of migration-stimulating factor (MSF), which they observed to be expressed in fetal fibroblasts but not adult fibroblasts (Chen et al., 1989). The data observed in this study appear to support these results, indicating that cardiac fibroblasts exhibit an increased migration rate at younger developmental ages, although this was not consistently observed as CFs did not show significant age-dependent differences in gelatin–fibronectin coatings.

The impact of cECM developmental age appeared most significant on CF cellular stiffness, particularly in the context of adult CFs, which showed an over two-fold increase in average cell stiffness when cultured on adult cECM compared to gelatin–fibronectin and fetal cECM (Figure 1). Differences in stiffness were less significant for neonatal and fetal CFs, regardless of cECM coating, apart from neonatal CFs cultured on fetal cECM, which had higher stiffness than neonatal CFs cultured on gelatin–fibronectin and adult cECM.

It has been established that CFs vary in their response to activation stimuli, including TGF-β1, matricellular proteins, and mechanical function, with fetal CFs exhibiting reduced, and often transient, activation compared to their adult CF counterparts (Souders et al., 2009; Ma et al., 2012; Avery et al., 2018; Cho et al., 2018). This provides a hypothesis for the differences we observed in cell stiffness and the migration rate, although to fully elucidate how developmental age drives differences in CF-ECM crosstalk, further studies directly targeting these stimuli are necessary to identify potential mechanisms.

### Activation metrics of CFs vary extensively with respect to the developmental age of both cECM and fibroblasts

Multiple avenues of research have been used to examine CF behavioral differences across the developmental age and have uncovered broad differences in how these fibroblasts mediate ECM regulation and wound healing. For example, fetal CFs have been established to promote the improved function of cardiomyocytes within engineered cardiac tissues compared to adult CFs, which appear to produce ECM proteins and cytokines that impede the function (Jonsson et al., 2016; Li et al., 2017). To the best of our knowledge, however, the impact of cECM developmental age on the behavior of cardiac fibroblasts, particularly with respect to CF developmental age, is not well-established.

This study reinforces the distinct differences observed in CFs at different developmental ages and offers insights into their cECM interactions. Imaging data on CF Ki67 and α-SMA expression indicated that the CF developmental age measurably altered CF response to cECM but that these changes were attenuated by TGF-β1 treatment. For example, without TGF-β1 treatment, neonatal CFs exhibited higher α-SMA expression than fetal and adult CFs on both fetal cECM and adult cECM. This contrast decreased slightly when CFs were treated with TGF-β1; however, neonatal CFs still presented greater α-SMA fluorescence on these coatings than fetal CFs, indicating age-distinct cell responses to these coatings (Figures 4A,B; Supplementary Table S1).

How cECM coating developmental age modulated cell activation was less conclusive, although fetal CFs had reduced α-SMA fluorescence on fetal cECM versus TCP, potentially suggesting a reduction in activation, although this was not observed when cells were activated with TGF-β1. Conversely, Ki67 evaluation to determine CF changes in proliferation was less conclusive, although TGF-β1-activated adult CFs exhibited a significantly higher fold change in Ki67 expression on TCP compared to other culture substrates (Figures 4C, D; Supplementary Table S1). However, these observations may be due, in part, to variations in cell density across developmental age conditions, which likely motivated potential proliferation.

Western blot analysis offered a more conclusive perspective on the impact of the cECM developmental age on CFs. Of note was the increased abundance of α-SMA in unactivated neonatal and adult CFs cultured on adult cECM compared to fetal cECM (Figure 5B), which suggested that the cECM developmental age may promote expression of this protein in CFs at these developmental stages. However, this difference was not observed in fetal CFs. Furthermore, the total SMAD2/3 content was elevated in neonatal CFs cultured on adult cECM (Figure 5C). Despite not showing significant differences with respect to the CF developmental age, these data support the idea that age-specific cECM differences might contribute to driving the CF phenotype. These observations could be due to age-dependent variation in ECM sensing due to integrin expression. Fetal dermal fibroblasts have been established to express different integrin receptors than adult fibroblasts (for example, α1 and α2 integrin subunits are downregulated in fetal fibroblasts) and exhibit less phenotypic change when cultured in monolayer compared to adult dermal fibroblasts (Chen et al., 1989; Zhang et al., 2001; Jonsson et al., 2016). Our previous work has also shown that fibroblasts and mesenchymal cells will respond to changing matrix composition via altered expression of integrin subunits (Gershlak and Black, 2015; Stoppel et al., 2016). A combination of developmental age-dependent phenotypes and altered integrin expression in response to ECM compositional differences is one possible mechanism through which the observed differences may occur.

However, it must be noted that significant differences in both α-SMA and SMAD expression were not observed with respect to either the CF or cECM developmental age when cells were treated with TGF-β1 (Figures 5B, C), potentially suggesting that this cytokine is more responsible for driving the CF phenotype than either the cell age or cECM developmental age. This is similar to the study by Cho *et al*, which concluded that TGF-β1 signaling dominated extracellular matrix rigidity as the driving factor in CF activation (Cho et al., 2018). From this conclusion, these results suggest that a fruitful area of future study will be investigating the potential of cECM at different developmental ages to mediate TGF-β1 availability in the microenvironment.

### Study limitations

Although this study indicates significant differences in the function of CFs with respect to the cell and cECM developmental age, some limitations must be addressed. In particular, conventional 2D cell culture is well-established to significantly impact the phenotype of cardiac fibroblasts. The stiff substrate provided by cell culture plastic has been shown to induce myofibroblast-like features in CFs, and extended *in vitro* culture similarly induces activation in CFs, necessitating the use of low-passage CFs to acquire the most physiologically relevant data (Mitchell et al., 2007; Hinz, 2009; Hinz, 2009; Souders et al., 2009). Therefore, it is likely that the 2D monolayer culture of the conducted experiments had some impact on the phenotype of the observed CFs. Additionally, the 2D culture system used in this work necessitated the use of pepsin-digested ECM, as opposed to whole ECM, which fragments available proteins and may liberate cell-instructive peptides. However, since cECM was fully digested, we believe that relative compositional differences are minimized and that previously reported differences in the cECM (Williams et al., 2014) are likely reflected in our data. Future work should recapitulate these experiments in a low-stiffness, 3D culture environment, potentially using undigested ECM, to confirm the results observed in 2D assays.

Although the Western blot assays to quantify levels of α-SMA and total SMAD2/3 protein expression in fibroblasts indicated that the variation in expression was evident with treatment and age, more work must be done to fully elucidate mechanistic changes in fibroblast activation that may occur due to ECM interactions. Specifically, phosphorylation of SMADs and translocation to the nucleus are well-established as necessary to enact a phenotypic change, and neither translocation nor PSMAD levels are explored in this study. However, we consider α-SMA and total SMAD2/3, which are correlated in previous cardiac studies (Hao et al., 2000; Ma et al., 2012; Khalil et al., 2017), to present valuable evidence that this line of inquiry is worthwhile to pursue in future studies.

It should also be noted that we do not account for fibroblast developmental heterogeneity in this study, evaluating them as a bulk cell population and, in the case of fetal and neonatal CFs, as a pooled population across the whole heart. Although CFs arising from different heart chambers possess important phenotypic differences (e.g., TGF-β1 signaling), a detailed evaluation of these differences was not feasible, given the technical challenges in isolating left ventricular tissue from fetal and neonatal rat hearts. Thus, further research evaluating differences in fibroblast populations and lineages with respect to the developmental age (Camelliti et al., 2005; Landry et al., 2019; Han and Zhou, 2022; Hall et al., 2023) and their interactions with ECM is a valuable future direction of this work.

## Conclusion

The objective of this study was to explore how cardiac fibroblasts vary in interactions with ECM across fetal, neonatal, and adult developmental ages and, similarly, to determine if the developmental age of cECM plays a role in the behavior of CFs *in vitro*. To this end, we evaluated CF behavior in 2D culture assays using fetal and adult solubilized porcine cECM coatings and determined that the cECM developmental age can modulate CF behavior and activation to a myofibroblast phenotype but that this effect is also dependent on the developmental age of the CFs and TGF-β1 treatment. The methods and results described herein contribute both to the overall knowledge of this diverse and critically important cell type and to the knowledge of cellular response to cECM materials, which are valuable as cardiac therapeutic and tissue engineering biomaterials. Future work must focus on further evaluation of the impact of the CF developmental age on their immune and inflammatory roles and reevaluating the results described herein in a 3D, physiologically relevant culture environment.

## Data Availability

The raw data supporting the conclusion of this article will be made available by the authors, without undue reservation.
