# Supplementary figures and images for: Characterization of cardiac fibroblast-extracellular matrix crosstalk across developmental ages provides insight into age-related changes in cardiac repair
Source: Front Cell Dev Biol. 2024 Feb 16;12:1279932. doi: 10.3389/fcell.2024.1279932 (PMC10904575; doi:10.3389/fcell.2024.1279932)

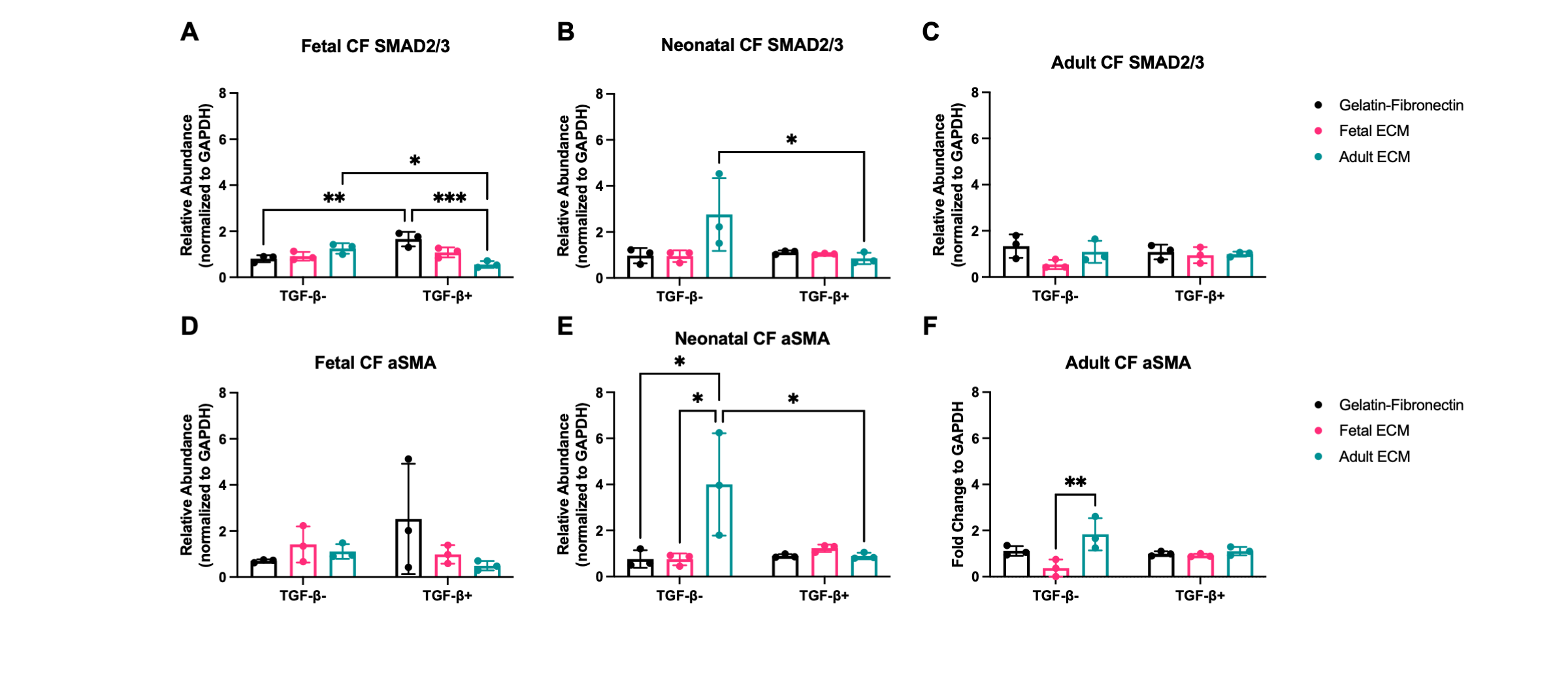

Supplement: Supplementary file 1 [file Image3.TIFF]

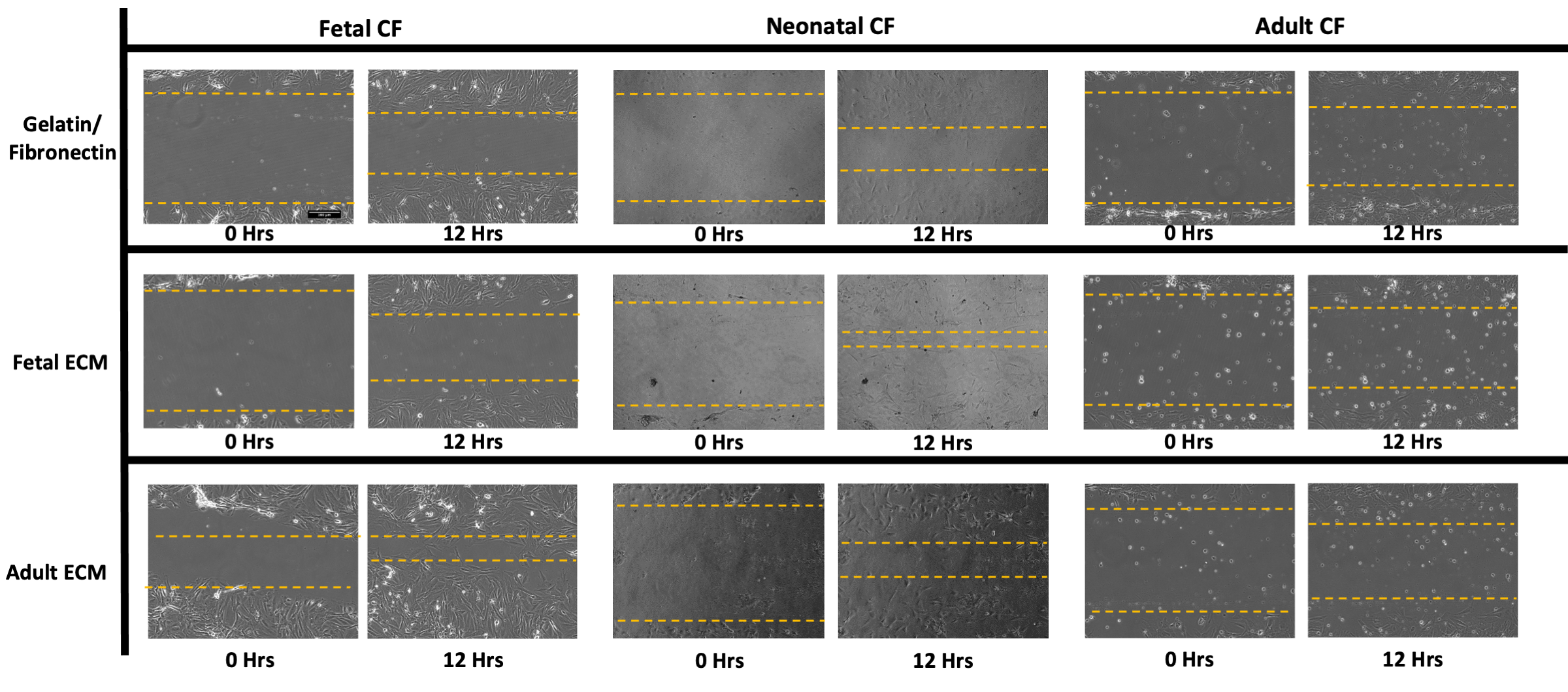

Supplement: Supplementary file 2 [file Image1.TIFF]

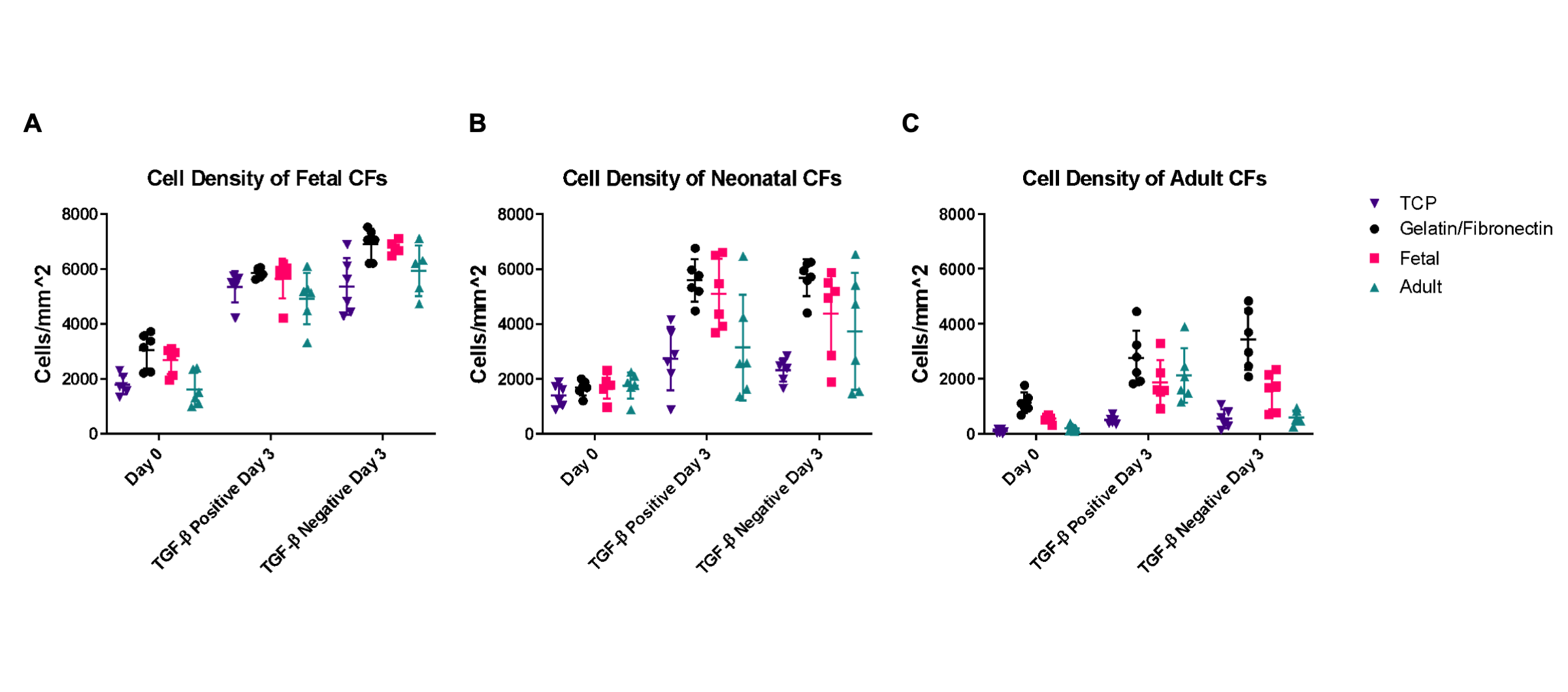

Supplement: Supplementary file 4 [file Image2.TIFF]
