# Supplementary material for: Characterization of cardiac fibroblast-extracellular matrix crosstalk across developmental ages provides insight into age-related changes in cardiac repair
Source: Front Cell Dev Biol. 2024 Feb 16;12:1279932. doi: 10.3389/fcell.2024.1279932 (PMC10904575; doi:10.3389/fcell.2024.1279932)
Supplement: Supplementary file 5 [file DataSheet1.docx]

**Characterization of cardiac fibroblast to extracellular matrix crosstalk across developmental age provides insight into age-related changes in cardiac repair**

**Luke R. Perreault ^1,$^, Mark Daley^1^, Matthew Watson^1^, Sagar Rastogi^1^, Ajith Jaiganesh^1^, Elizabeth Porter^2^, Breanna Duffy^1^, Lauren D. Black III^1,2^**

^1^ Department of Biomedical Engineering, Tufts University, Medford, MA 02155

^2^ Cellular, Molecular and Developmental Biology Program, Graduate School for Biomedical Sciences, Tufts University School of Medicine, Boston, MA 02111

^$^ Current Affiliation, Department of Engineering, Boston College, Chestnut Hill, MA 02467

**Captions for Supplementary Materials**

**Supplemental Figure 1** - Representative images of all scratch assay conditions (cell/ECM ages) at T=0 and T=12 hours. Yellow dotted lines denote cell-free region in all images.

**Supplemental Figure 2** - Cell density (in cells/mm^2^) image quantification of Day 0 (pre-treatment) and Day 3 fetal, neonatal, and adult cardiac fibroblasts when cultured on gelatin-fibronectin, fetal cECM, adult cECM, and TCP with A) showing fetal CF cell density across all conditions, and either treated or untreated with TGF-β1, and B) and C) showing neonatal and adult CF cell density under the same conditions.

**Supplemental Figure 3** - Separate western blot analysis of fetal, neonatal, and adult cardiac fibroblasts for total SMAD2/3 and α-SMA at Day 3 of culture, either treated with TGF-β1 or untreated, on gelatin-fibronectin, fetal cECM, or adult cECM coatings. A), B), and C): relative abundance of total SMAD2/3 normalized to GAPDH for fetal CFs, neonatal CFs, and adult CFs, respectively. D), E), and F): relative abundance of α-SMA normalized to GAPDH for fetal CFs, neonatal CFs, and adult CFs, respectively. N=3, all groups. * = P<0.05, ** = P<0.01, *** = P<0.001.

**Supplementary Table 1** – Table of comparisons for image analysis data, reporting CF-to-CF and ECM-to-ECM comparisons of D3 fetal, neonatal, and adult cardiac fibroblasts when cultured on gelatin-fibronectin, fetal ECM, adult ECM, and TCP. N>5, all groups. N>5, all groups. * = P<0.05, ** = P<0.01, *** = P<0.001, **** = P<0.0001, NS = no significance.
